# Maternal sodium butyrate supplement elevates the lipolysis in adipose tissue and leads to lipid accumulation in offspring liver of weaning-age rats

**DOI:** 10.1186/s12944-016-0289-1

**Published:** 2016-07-22

**Authors:** Jiabin Zhou, Shixing Gao, Jinglong Chen, Ruqian Zhao, Xiaojing Yang

**Affiliations:** Key Laboratory of Animal Physiology & Biochemistry, Nanjing Agricultural University, Nanjing, 210095 People’s Republic of China; Jiangsu Collaborative Innovation Center of Meat Production and Processing, Quality and Safety Control, Nanjing, 210095 People’s Republic of China

**Keywords:** Maternal sodium butyrate, Maternal lipolysis, Fatty acid transport, Offspring lipid metabolism

## Abstract

**Background:**

Sodium butyrate (SB) is reported to regulate lipid metabolism in mammals, and the relationship between maternal nutrition and offspring growth has drawn much attention in the last several years.

**Methods:**

To elucidate the effects of maternal dietary SB supplementation on hepatic lipid metabolism in weaning rats, we fed 16 primiparous purebred female SD rats either a chow-diet or a 1 % sodium butyrate diet throughout pregnancy and lactation. At weaning age, samples of the maternal subcutaneous adipose tissue and offspring liver were taken. The serum indexes and expressions of proteins related to lipid metabolism were detected in the mother and offspring, respectively.

**Results:**

The results showed that the maternal SB supplement increased the concentration of non-esterified fatty acid (NEFA) in the maternal and offspring serum (*P* < 0.05). Total cholesterol (Tch) increased significantly in the weaning-rat serum (*P* < 0.05). Maternal adipose tissue from the SB-supplemented rats showed higher content of protein G-coupled protein (GPR43) and protein kinase A (PKA) (*P* < 0.05). The expression of protein adipose triglyceride lipase (ATGL), and of total and phosphorylated hormone sensitive lipase (HSL), in the maternal adipose tissue increased significantly (*P* < 0.05) compared to the control group. However the proteins related to lipogenesis showed no significant changes. Moreover, the concentration of triglyceride in the offspring liver increased significantly, and this likely resulted from an increase in the levels of fatty acids binding protein (FABP) and fatty acid translocase (CD36) protein (*P* < 0.05). SB exposure during pregnancy and lactation increased the hepatic total cholesterol (Tch) content (*P* < 0.01), which was related to a significantly up-regulated offspring hepatic expression of low density lipoprotein receptor (LDLR) protein (*P* < 0.05).

**Conclusion:**

These results indicate that a maternal SB supplement during pregnancy and the lactation period promotes maternal fat mobilization, which may result in fatty acid uptake and lipid accumulation in the liver of the offspring.

## Background

Lines of evidence indicate that maternal nutrition during pregnancy and lactation is closely related to the adequate development of offspring [1]. The offspring’s metabolism is dependent on substrates from maternal nutrients, and one of the most important nutritional substances is fatty acid (FA) [2]. FAs are responsible for the growth of cell membranes, as well as for maintaining their appropriate fluidity and permeability, [3] and they are involved in energetic and metabolic processes [4]. The maternal FA level is positively related to the offspring’s fat percentage, and transporting FAs from mother to offspring is positively correlated with offspring development, particularly during the late gestation period, when deposition of fat in the fetus increases sharply [5]. It has been shown that maternal triglycerides (TG) and free fatty acid levels have a close relationship with both weight and fat mass of infants [3]. FA deficiency and disruption in the maternal-placental offspring metabolism lead to malnutrition of the fetus, and metabolic diseases may appear in the later life of the offspring [6, 7].

Short chain fatty acids (SCFA) can be found in foods and are the main products of the intestinal fermentation of soluble fiber, which contains abundant quantities of acetate, propionate and butyrate [8, 9]. Butyrate acts as the major intestinal fuel, supplying 60–70 % of the energy needs of the colonocytes [10, 11]. Increasing evidence have shown that butyrate affects body lipid metabolism [12–14]. Several studies have discussed the treatment of butyrate reduced intracellular lipolytic activity or enhanced adipogenesis [11, 15]. Other reports have suggested that incubation of 3 T3-L1 adipocytes with high concentrations of butyrate resulted in an increased lipolytic response [16]. In a high-fat animal model, treatment with sodium butyrate (SB) can lead to loss of body weight or reverse gains in body weight and adiposity [17, 18].

Regarding supplemental SB in the maternal diet, lines of evidence have indicated that SB can promote the growth performance of weaning pigs [19–21]. However, the effect of a maternal SB supplement on the maternal lipid metabolism is unknown. Furthermore, if SB influences the mother’s lipid metabolism, it remains largely unclear whether this effect will exert its action on offspring lipid accumulation.

The liver performs a key role in lipid metabolism, importing free fatty acids, manufacturing and exporting lipids to provide energy, and storing excess lipids, thus making the liver responsible for lipid homeostasis [22]. It has been shown that maternal pre-pregnancy body weight is associated with offspring hepatic fat storage [23]. Maternal obesity may result in offspring overgrowth and increased lipid deposition in fetuses [24]; moreover, an excess of lipid deposition can lead to postnatal hepatic dysfunction and even non-alcoholic steatohepatitis [25]. The influence of maternal SB on offspring hepatic lipid metabolism needs to be investigated.

We conducted this study to determine the effects of maternal butyrate supplementation on the potential relationship between maternal lipid metabolism in white adipose tissue and offspring hepatic lipid accumulation during pregnancy and the lactation period. This is the first study to examine whether a relationship exists between maternal serum NEFA and offspring hepatic lipid accumulation. Overall, our results will provide scientific research to help protect offspring liver from damage.

## Methods

### Animals and treatments

Twenty-four ten week old female virgin SD rats were purchased from the experimental animal center of Jiangsu University. All rats were housed in pairs at 20–24 °C and 40–60 % humidity on a 12 h light–dark cycle with adlibitum access to food and water.

After one week of acclimatization, the rats were randomly divided into a 1:2 ratio. All rats received a normal chow diet (20 % protein) until pregnancy was confirmed through the presence of a post-copulatory plug and vaginal smear the next morning. All of the males were removed after the females became pregnant. The pregnant rats were fed either a normal diet or a 1 % sodium butyrate diet until the end of the lactation period. The above foods were all customized from the Jiangsu Xie Tong Company. SB was blended into the normal diet as it was formed, and the food was stored at 4 °C after it was pelleted.

The slaughter and sampling procedures complied with the “Guidelines on Ethical Treatment of Experimental Animals” (2006) No.398 set by the Ministry of Science and Technology, China.

### Blood and tissue sample collection

At 21 days of age, the weaning rats and mothers were weighed and anesthetized with an intraperitoneal injection of Nembutal, and then they were euthanized. Blood was drawn from the abdominal aorta and centrifuged at 3000 rpm for 10 min at 4 °C for serum collection. Serums were stored at −20 °C until they were analyzed. The fresh livers from the weaning rats and the subcutaneous adipose tissue derived from the mothers were immediately removed and weighed after laparotomy. The samples were snap-frozen in liquid nitrogen and stored at −80 °C.

### Assay of serum concentration of cholesterol and hepatic contents of cholesterol and fatty acids

Serum concentration of total cholesterol (Tch) was measured with a biochemical automatic analyzer (Hitachi 7020; HITACHI) using a commercial cholesterol assay kit (E1015; Applygen Technologies, Inc.). Serum concentrations of LDL-cholesterol (LDL-C) and HDL-cholesterol were measured with a biochemical automatic analyzer (Hitachi 7020; HITACHI) using a commercial cholesterol assay kit (KP712, KF253 Wako Pure Chemical Industries, Ltd. Wako), respectively. Serum concentration of TG was measured with a biochemical automatic analyzer (Hitachi 7020; HITACHI) using a commercial TG assay kit (E1013; Applygen Technologies, Inc.). Serum non-esterified fatty acid (NEFA) concentrations were determined using the Wako NEFA Cacyllcoenzyme A synthetase acyl-coenzyme A oxidase assay method. Hepatic total cholesterol concentration was measured using a tissue total cholesterol assay kit (E1015; Applygen Technologies, Inc.) following the manufacturer’s instructions. Hepatic concentration of total triglyceride was quantified with a biochemical automatic analyzer (Hitachi 7020; HITACHI) using a commercial TG assay kit (E1013; Applygen Technologies, Inc.).

### Assay of maternal serum concentration of fatty acid composition

Serum fatty acids (FAs) composition was analyzed according to the method of Hossain Z [26]. Briefly, total lipids from the serum were extracted with chloroform-methanol (2:1, vol:vol) containing 0.01 % butylated hydroxytoluene (Sigma-Aldrich) by using heptadecanoic acid as an internal standard (Sigma-Aldrich). The fatty acid extracts were methylated with methanolic hydrochloric acid. The fatty acid methyl esters were analyzed by an Agilent 6890 N gas chromatograph with a flame ionization detector (Agilent Technologies) as mentioned. Serum FAs are shown as percentage (%, wt/wt) composition. The gas chromatograph was equipped with a split/split less injector, and the column was a fused silica DB-225MS capillary column (30 m × 0.25 mm thickness; Supelco). The gas chromatograph oven was programmed as follows: 70 °C for 2 min, increase 25 °C/min, 190 °C for 3 min, increase 2 °C /min, 220 °C for 15 min, increase 45 °C, and finally 250 °C for 10 min. Samples were run with a 5:1 split ratio, and helium was used as the carrier gas with a column flow rate of 0.8 ml/min. Temperature for the injector was operated at 280 °C. Temperature for the combustion reactor was set at 960 °C. The fatty acids were determined by comparing the peak retention times with standards (Sigma, St Louis, MO).

### Protein extraction and Western blot analysis

Total proteins were extracted from 60 mg of frozen liver samples as described previously. Protein concentrations were determined using a Pierce BCA Protein Assay kit (Pierce, Rockford, IL, USA). 48 μg of liver protein was then loaded onto 10 % or 15 % SDS-PAGE gel. Western blot analysis of target proteins was carried out according to the protocols provided by the manufacturer. The primary antibodies used in the Western blot analysis are listed in Table 1. GAPDH or β-actin was used as the loading control in the Western blot analysis.

**Table 1:** Antibodies used in our experiment

Antibody description	Company	Item No.	Dilution ratio
ACSL1	santa cruz	sc-49008	1:200
ACSS1	santa cruz	sc-373847	1:200
SCD1	santa cruz	sc-14720	1:200
PPARγ	bioworld	BS-1587	1:500
SREBP1	santa cruz	sc-366	1:200
HSL	bioworld	BS2742	1:500
P-HSL	santa cruz	sc-139656	1:500
ATGL	bioworld	BS6757	1:500
CPT1α	abcam	ab83863	1:500
UCP3	bioworld	BS6757	1:500
GPR43	santa cruz	sc-32906	1:200
PKA	bioworld	BS2648	1:500
SREBP2	santa cruz	sc-13068	1:200
HMGCR	bioworld	BS6625	1:500
CYP27A1	bioworld	bs2192	1:200
LXR	santa cruz	sc-13068	1:200
LDLR	proteintech	10785-1-AP	1:1000
C/EBPβ	santa cruz	sc-150×	1:200
FABP1	bioworld	BS7533	1:500
CD36	bioworld	BS7861	1:500
GAPDH	bioworld	MB001H	1:10000
β-actin	bioworld	AP0060	1:10000

### Statistical analyses

The results from the experiment were analyzed using the statistics software SPSS for Windows version 20.0. Independent-samples *T*-Test was used to analyze all data. The results were presented as the mean ± standard error (SE). Differences were considered statistically significant when the p value was less than 0.05. Numbers of replicates used in the experiment were noted in the tables and figures.

## Results

### Body weight, liver weight and litter size

The mother’s body weight showed no significant changes between the Control (Con) group (315.16 ± 9.67) and the SB group (376.56 ± 9.18). The litter weight in the Con group (102.00 ± 2.92) was not significant compared to the SB group (107.03 ± 3.20). The litter size in the Con (*n* = 6) was (13.85 ± 0.34) compared to the SB group (16.00 ± 0.53). The offspring weight and liver weight in the Con group in the weaning rats were comparable to the SB group (*P* < 0.05), as is shown in Table 4.

### Maternal serum NEFA, triglyceride, lipoprotein lipase and cholesterol concentration

As is shown in Table 2, serum content of NEFA was significantly increased (*P* < 0.05) in the sodium butyrate–supplemented rats compared with the Con group, though the maternal serum Tch, TG, LDLC and HDLC showed no obvious changes. The serum concentrations of LPL in the mothers also demonstrated no difference.

**Table 2:** Serum concentration of total cholesterol, triglyceride and non-esterified fatty acid in maternal rats (*n* = 6)

Variables	Con	SB	*P*-value
Tch (mmol/L)	1.84 ± 0.18	1.60 ± 0.10	0.77
TG (mmol/L)	0.48 ± 0.07	0.52 ± 0.06	0.68
HDL-C (mmol/L)	1.06 ± 0.08	0.95 ± 0.08	0.50
LDL-C (mmol/L)	0.19 ± 0.02	0.15 ± 0.01	0.92
LPL (g/L)	12.00 ± 0.87	10.71 ± 1.13	0.59
NEFA (mmol/L)	0.68 ± 0.05	0.86 ± 0.05	0.03

Tch, total cholesterol; LDL-C, LDL-cholesterol; HDL-C, HDL-cholesterol; LPL, lipoprotein lipase, NEFA, non-esterified fatty acids

### The fatty acid composition of the maternal serum

The fatty acid composition of the maternal serum is shown in Table 3. The other fatty acid components tested did not change significantly, except the Cis-9-Oleic Methyl ester (c18:1n9c), which increased significantly compared to the Con group.

**Table 3:** The non-esterified fatty acid composition in maternal serum (in % of total fatty acids)

Variables	Con	SB	*P*-value
c6:0	40.7 ± 3.6	30.9 ± 6.6	0.20
c14:1	12.0 ± 0.7	14.7 ± 2.1	0.21
C18:0	9.6 ± 2.0	11.0 ± 2.5	0.69
c18:1n9c	14.3 ± 2.2	22.9 ± 3.1	0.05
c18:2n6c	11.6 ± 0.2	10.1 ± 1.3	0.31
c20:4n6	5.8 ± 1.2	3.1 ± 0.8	0.10

c6:0, caproic acid; c14:1, myristoleic acid; C18:0, stearic acid; c18:1n9, oleic acid;c18:2n6c, linoleic acid; c20:4n6, arachidontic acid

### The concentration of Tch and TG in offspring liver and serum

As is shown in Table 4, the serum content of NEFA was significantly increased (*P* < 0.05) in the sodium butyrate-supplemented rats. No significant changes in concentrations of TG were observed in the offspring serum. However, the offspring serum content of total cholesterol, LDL-C and HDL-C were significantly increased (*P* < 0.01) compared to the Con group. Moreover, the hepatic concentration of TG and Tch in offspring born to the sodium butyrate supplemented group increased significantly (*P* < 0.05).

**Table 4:** Serum concentration of fatty acids, total cholesterol, triglyceride and hepatic content of total cholesterol, triglyceride in weaning rats, body weight and liver weight

Variables	Con	SB	*P*-value
Body weight (g)	68.51 ± 1.64	67.67 ± 3.06	0.81
Liver weight (g)	3.20 ± 0.13	3.18 ± 0.18	0.80
HepaticTch (mmol/L)	4.39 ± 0.07	5.42 ± 0.30	0.00
HepaticTG (mmol/L)	15.83 ± 1.17	22.72 ± 2.57	0.04
Serum Tch (mmol/L)	2.88 ± 0.09	3.43 ± 0.14	0.00
Serum TG (mmol/L)	0.76 ± 0.14	0.76 ± 0.20	1.00
Serum HDL-C (mmol/L)	1.20 ± 0.03	1.50 ± 0.07	0.00
Serum LDL-C (mmol/L)	0.99 ± 0.06	1.25 ± 0.07	0.01
Serum NEFA (mmol/L)	0.51 ± 0.05	0.65 ± 0.04	0.05

Tch, total cholesterol; LDL-C, LDL-cholesterol; HDL-C, HDL-cholesterol; NEFA, non-esterified fatty acid

(Mean values with their standard errors, *n* = 6)

### Expression of proteins related to lipid metabolism in the maternal subcutaneous adipose tissue

**Fig. 1:**
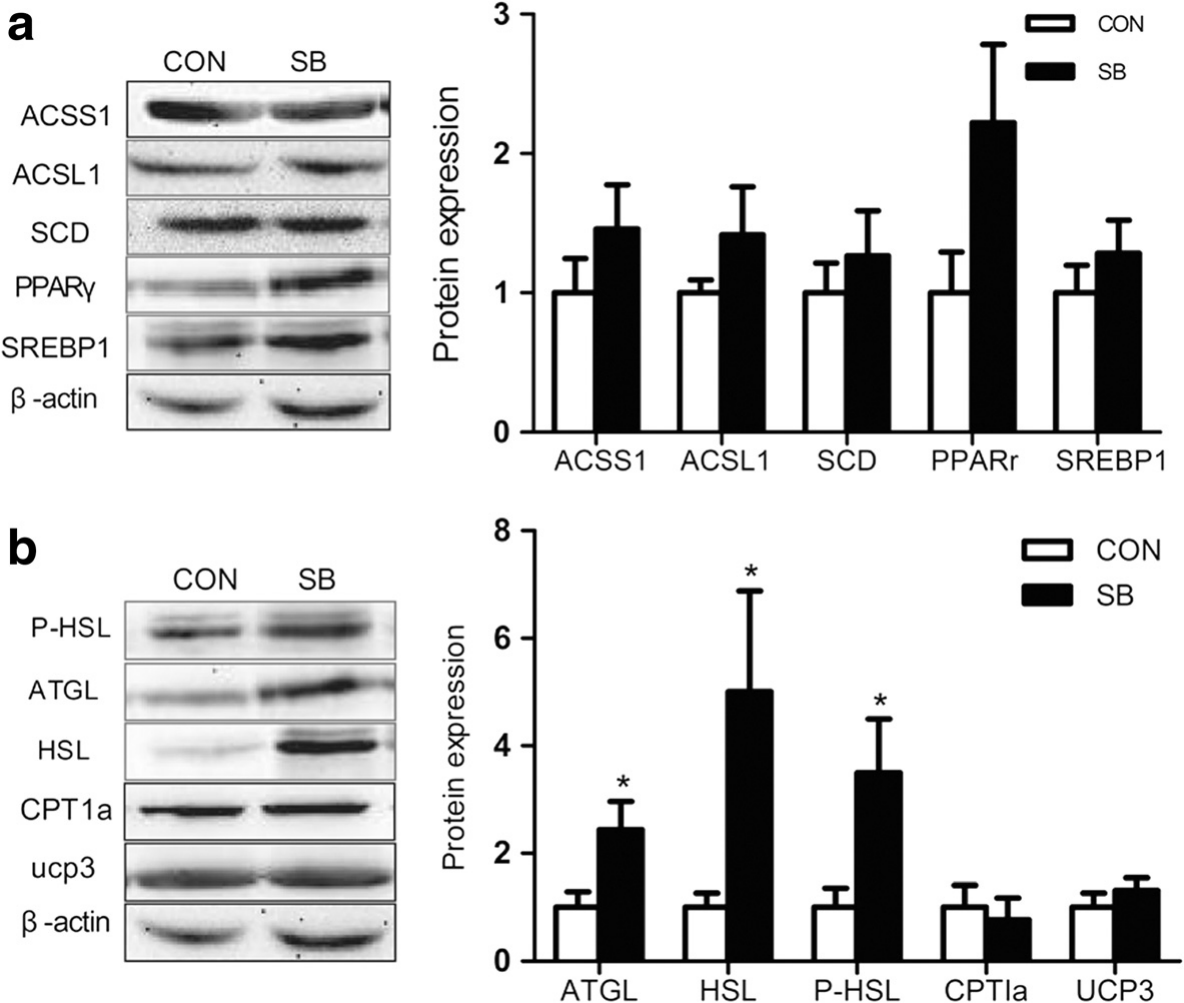
Effect of maternal sodium butyrate on lipid metabolism in maternal adipose tissue. **a** The expression of proteins related to lipogenesis. **b** The expression of proteins involved in lipolysis. Values are mean ± SEM, *P < 0.05, compared with control (*n* = 6)

**Fig. 2:**
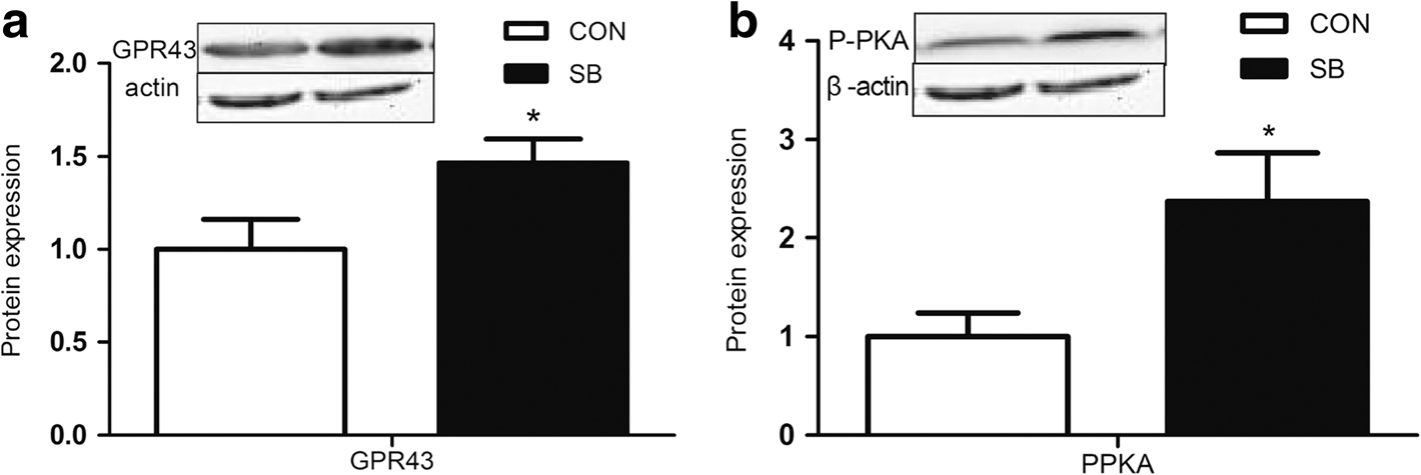
The GPR43 (**a**) and PKA (**b**) protein content in maternal adipose tissue. Values are mean ± SEM, *P < 0.05, compared with control (*n* = 6)

As is shown in Fig. 1, the proteins involved in lipolysis, including ATGL, HSL and P-HSL were significantly higher in the SB group (*P* < 0.05) (Fig. 1b). Yet, no significant changes were detected for the content of proteins long-chain acyl-CoA synthetase-1 (ACSL1), Acyl-CoA Synthetase-1 (ACSS1), stearyl coenzyme A dehydrogenase (SCD) (Fig. 1a), or in the content of SREBP1c and PPARγ in the subcutaneous adipose tissue (Fig. 1a). Moreover, the protein content of carnitine palmitoyltransferase (CPTI-α) 1A and UCP3uncoupling protein 3 involved in energy metabolism did not change in the SB group compared with the Con group (Fig. 1b). In addition, the content of proteins GPR43 and PKA were significantly higher in the SB group (*P* < 0.05) (Fig. 2).

### Expression of proteins related to lipid metabolism in the offspring liver

**Fig. 3:**
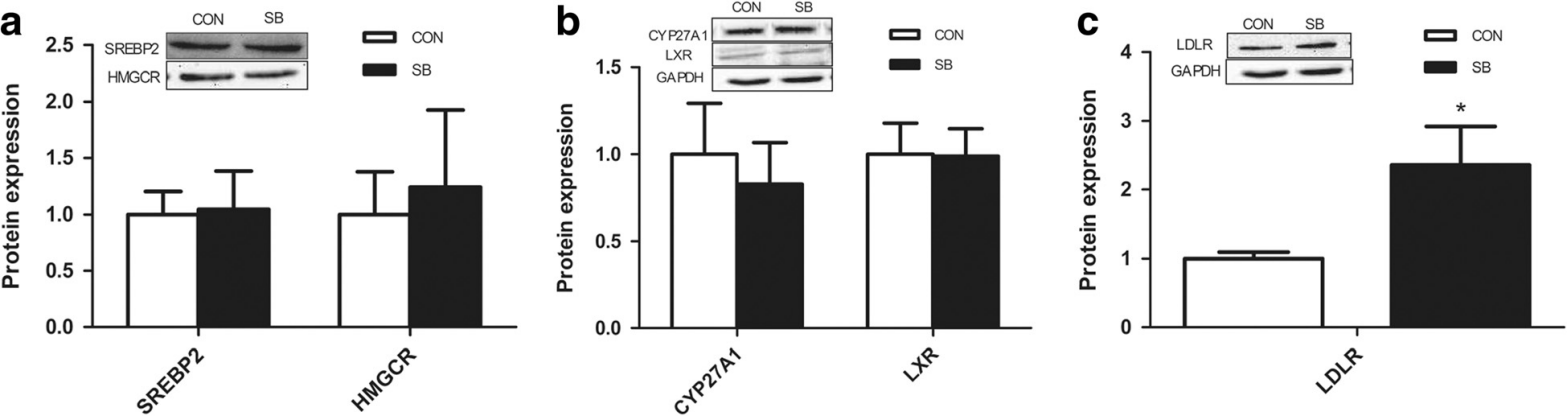
Effect of maternal sodium butyrate on hepatic expression of cholesterol metabolic proteins in weaning rats. **a** Protein expression of SREBP2 and HMGCR; **b** Protein expression of CYP27A1 and LXR; **c** Protein expression of LDLR. Values are mean ± SEM, *P < 0.05, compared with control (*n* = 6)

The results showed that the LDLR protein active in cholesterol uptake increased significantly in the SB group compared with the Con group (Fig. 3c). While, the proteins expression involved in cholesterol synthesis, including 3-hydroxy-methyl-glutaryl-CoA reductase (HMGCR) and Sterol Regulatory Element Binding Protein2 (SREBP2) showed no significant changes (Fig. 3a). Additionally, the proteins related to cholesterol degradation, such as sterol 27-hydroxylase (CYP27A1), liver X-activated receptor (LXR) (Fig. 3b) remained unchanged compared to the Con group.

**Fig. 4:**
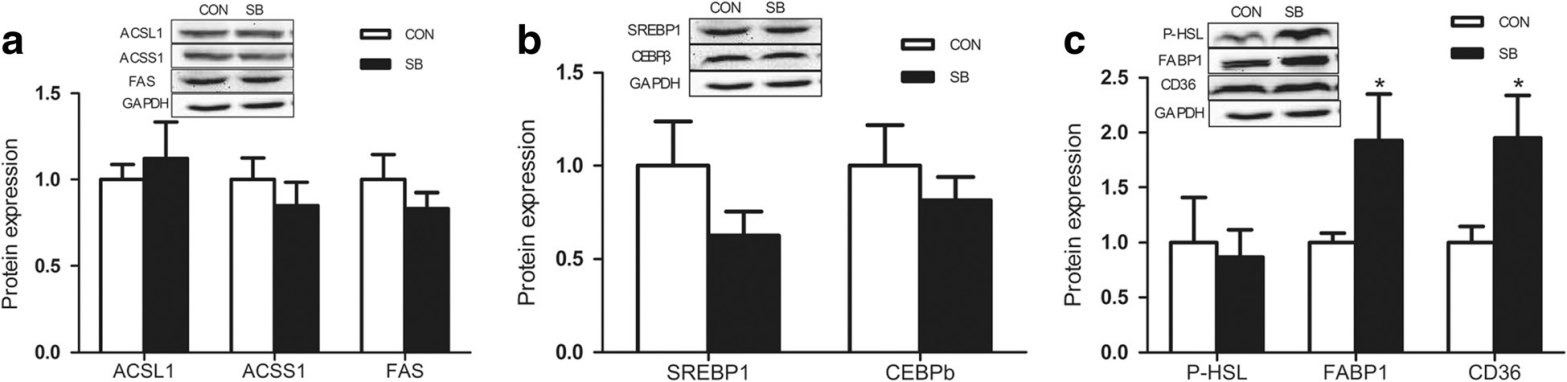
Effect of maternal sodium butyrate on hepatic expression of lipid metabolic proteins in weaning rats. **a** Proteins involved in lipogenesis; **b** Protein expression of SREBP1 and CEBP-b. **c** Protein expression of P-HSL,FABP1 and CD36. Values are mean ± SEM, *P < 0.05, compared with control (*n* = 6)

The protein content of FABP1 and CD36 related to fatty acid transport in the offspring liver increased significantly (Fig. 4c), though proteins related to TG synthesis such as ACSS1, ACSL1, FAS (Fig. 4a), sterol regulatory element binding protein 1 (SREBP1) and CCAAT-enhancer binding proteins (CEBPβ) (Fig. 4b) were the same in both groups. Meanwhile, no alterations were detected in the content of P-HSL protein levels in two groups (Fig. 4c).

## Discussion

It is well established that resistant starch and dietary fibers can produce SCFA, which can raise intestinal and circulating SCFA, including butyrate acid. Besides acting as an important energy source of the colonocytes, butyrate is of special interest for its countless positive effects on the gut and other tissues [27]. In a rodent obesity model, both supplementation with butyrate and oral administration of acetate have been demonstrated to suppress weight gain [12]. In a maternal diet, supplementing with butyrate has been shown to increase reproductive performance and enhanced anti-oxidant capacity [28]. In the present study, we demonstrated for the first time (as far as we know) the influence of a butyrate supplement on maternal and offspring lipid metabolism.

The present study demonstrated that the increasing concentration of serum NEFA in maternal serum was consistent with the increasing lipolysis in the adipose tissue. The higher protein expressions of ATGL, HSL and P-HSL were observed in maternal adipose tissue from the SB group. ATGL could stimulate lipid mobilization when it was localized at the lipid droplet surface, and it could catalyze the original step in TG hydrolysis [29, 30]. HSL was thought to be the rate-limiting enzyme of the first lipolytic step [30, 31]. Moreover, previous studies have shown that oleic acid has a potentially protective effect on cancers and cell growth [32]. Similarly, an increased composition of oleic acid was found in our present study. Meanwhile, we investigated the protein levels involved in lipogenesis, including ACSL1, ACSS1, SCD and the key nuclear transcription factors containing PPARγ and SREBP1; none of these showed obvious changes.

GPR43 is one of the FA receptors and is present in many different kinds of tissues, including adipose tissue. In order to understand the relationship between sodium butyrate and maternal lipolysis, we determined the GPR43 protein expression in the maternal adipose tissue. Notably, the GPR43 protein level was elevated in the present study. It has been shown that butyrate can activate PKA through cAMP after binding the receptor [33], and other research has demonstrated that PKA can activate and phosphorylate the downstream molecules of HSL [33–35]. In the present study, the results showed that the PKA protein level increased significantly in the SB group. All of our results taken together showed that sodium butyrate increased the protein expression of GPR43, which elevated the P-HSL through the higher PKA protein level.

To further clarify the effect of a maternal sodium butyrate supplement on offspring lipid metabolism, we measured the serum lipid indexes in the offspring. Our results found that the concentration of serum NEFA in the SB group is significantly higher than in the Con group. When this result is considered in association with the increased maternal serum NEFA, it may be inferred that the enhanced maternal serum NEFA may have increased the transport capacity in the placenta and resulted in the increased serum NEFA in the offspring. Also, there is a report exhibiting that a maternal SB supplement can increase the fatty acid transport in the placenta [36]. However, we supplemented the SB during gestation and the lactation period; therefore, it is also possible that the maternal NEFA was transported through the milk [37]. Further study is needed to clarify this issue.

The liver is a main actor in the process of fatty acid metabolism, and the present results showed a higher hepatic content of TG and Tch after maternal butyrate treatment. We further investigated the expression of lipid metabolism related proteins in the offspring liver. Previous studies have suggested that the fatty acid-binding protein known as FABP1 is present at high levels in murine liver [38], and it is a key regulator of hepatic lipid metabolism and acts as an intracellular acceptor of fatty acids following their cellular uptake and trafficking [39]. The increased FABP1 protein expression in the offspring liver in the present study indicates a higher uptake of free fatty acids, and these findings are consistent with the higher concentration of NEFA in the offspring serum. The main membrane proteins associated with FABPs are the fatty acid transfer proteins (FAT/CD36 and FATP) [40]. We thus found a significant up-regulation of CD36 protein levels in the offspring liver of the maternal sodium butyrate group. Another important finding of the present study is the higher LDLR protein levels in the offspring liver of those exposed to the maternal sodium butyrate supplement. LDL particles are the main carrier of cholesterol to peripheral tissues in mammals, and the hepatic LDLR is important for maintaining cholesterol homeostasis [41]. These results are in agreement with the elevated Tch in the offspring liver treated with maternal sodium butyrate. However, the other proteins related to lipogenesis and lipolysis detected in the present study showed no obvious changes compared with the control group. It is possible that the increased offspring hepatic lipid deposition in the maternal SB group resulted mainly from the increased hepatic lipid uptake from the circulating serum.

## Conclusion

In summary, SD rats treated with maternal sodium butyrate throughout gestation and lactation showed increased lipolysis in the maternal adipose tissue and in lipid accumulation in the offspring liver. Follow-up studies are needed to identify the possible pathway of fatty acid transport between mother and offspring after the maternal sodium butyrate supplement was administered.

## Abbreviations

ACSL1, long-chain acyl-CoA synthetase-1; ACSS1, Acyl-CoA Synthetase-1; ATGL, adipose triglyceride lipase; CD36, fatty acid transfer proteins; CEBPβ, CCAAT-enhancer binding proteins; CON, control; CPT-1A, Carnitine palmitoyltransferase 1A; CYP27A1, sterol 27-hydroxylase; FABP1, fatty acid binding proteins; FAS, fatty acid synthetase; GAPDH, glyceraldehyde-3-phosphate dehydrogenase; GPR43, G-coupled protein receptor 43; HSL, hormone sensitive lipase; LDL-R, Low-density lipoprotein receptor; LXR, liver X-activated receptor; NEFA, non esterified fatty acid; p-HSL, phosphorylated-hormone sensitive lipase; PKA, proteinkinase A; PPARγ, peroxisome proliferative activated receptor γ; SB, sodium butyrate; SCD, Stearoyl-CoA desaturase; SREBP1, sterol regulatory element binding protein 1; Tch, total cholesterol; TG, triglyceride; UCP3, Uncoupling protein 3

## References

[1] Islam A, Kagawa Y, Sharifi K, Ebrahimi M, Miyazaki H, Yasumoto Y, Kawamura S, Yamamoto Y, Sakaguti S, Sawada T (2014). Fatty Acid Binding Protein 3 Is Involved in n-3 and n-6 PUFA transport in mouse trophoblasts. J Nutr.

[2] Diaz P, Harris J, Rosario FJ, Powell TL, Jansson T (2015). Increased placental fatty acid transporter 6 and binding protein 3 expression and fetal liver lipid accumulation in a mouse model of obesity in pregnancy. Am J Physiol Regul Integr Comp Physiol.

[3] Larque E, Ruiz-Palacios M, Koletzko B (2013). Placental regulation of fetal nutrient supply. Curr Opin Clin Nutr Metab Care.

[4] Haggarty P (2004). Effect of placental function on fatty acid requirements during pregnancy. Eur J Clin Nutr.

[5] Brett KE, Ferraro ZM, Yockell-Lelievre J, Gruslin A, Adamo KB (2014). Maternal-fetal nutrient transport in pregnancy pathologies: the role of the placenta. Int J Mol Sci.

[6] Fowden AL, Ward JW, Wooding FP, Forhead AJ, Constancia M (2006). Programming placental nutrient transport capacity. J Physiol.

[7] Barker DJ, Bagby SP, Hanson MA (2006). Mechanisms of disease: in utero programming in the pathogenesis of hypertension. Nat Clin Pract Nephrol.

[8] Jin CJ, Sellmann C, Engstler AJ, Ziegenhardt D, Bergheim I (2015). Supplementation of sodium butyrate protects mice from the development of non-alcoholic steatohepatitis (NASH). Br J Nutr.

[9] Nicholson JK, Holmes E, Kinross J, Burcelin J, Gibson G, Jia W, Pettersson S (2012). Host-Gut Microbiota Metabolic Interactions. Science.

[10] Hong YH, Nishimura Y, Hishikawa D, Tsuzuki H, Miyahara H, Gotoh C, Choi KC, Feng DD, Chen C, Lee HG (2005). Acetate and propionate short chain fatty acids stimulate adipogenesis via GPCR43. Endocrinology.

[11] Ge H, Li X, Weiszmann J, Wang P, Baribault H, Chen JL, Tian H, Li Y (2008). Activation of G protein-coupled receptor 43 in adipocytes leads to inhibition of lipolysis and suppression of plasma free fatty acids. Endocrinology.

[12] Gao Z, Yin J, Zhang J, Ward RE, Martin RJ, Lefevre M, Cefalu WT, Ye J (2009). Butyrate improves insulin sensitivity and increases energy expenditure in mice. Diabetes.

[13] Ohira H, Fujioka Y, Katagiri C, Mamoto R, Aoyama-Ishikawa M, Amako K, Izumi Y, Nishiumi S, Yoshida M, Usami M, Ikeda M (2012). Butyrate attenuates inflammation and lipolysis generated by the interaction of adipocyte and macrophages. J Atheroscler Thromb.

[14] Lin HV, Frassetto A, Kowalik EJ, Nawrocki AR, Lu MM, Kosinski JR, Hubert JA, Szeto D, Yao X, Forrest G, Marsh DJ (2012). Butyrate and propionate protect against diet-induced obesity and regulate gut hormones via free fatty acid receptor 3-independent mechanisms. PLoS ONE.

[15] Yan H, Ajuwon KM (2015). Mechanism of Butyrate Stimulation of Triglyceride Storage and Adipokine Expression during Adipogenic Differentiation of Porcine Stromovascular Cells. PLoS ONE.

[16] Rumberger JM, Arch JR, Green A (2014). Butyrate and other short-chain fatty acids increase the rate of lipolysis in 3 T3-L1 adipocytes. Peer J.

[17] Den Besten G, Aycha B, Albert G, Van Eunen K, Rick H, Van Dijk TH, Oosterveer MH, Jonker JW, Groen AK, Dirk-Jan R (2015). Short-Chain Fatty Acids protect against High-Fat Diet-Induced Obesity. Diabetes.

[18] Frost G, Sleeth ML, Sahuri-Arisoylu M, Lizarbe B, Cerdan S, Brody L, Anastasovska J, Ghourab S, Hankir M, Zhang S (2014). The short-chain fatty acid acetate reduces appetite via a central homeostatic mechanism. Nat Commun.

[19] Le Gall M, Gallois M, Seve B, Louveau I, Holst JJ, Oswald IP, Lalles JP, Guilloteau P (2009). Comparative effect of orally administered sodium butyrate before or after weaning on growth and several indices of gastrointestinal biology of piglets. Br J Nutr.

[20] Gálfi P, Bokori J (1990). Feeding trial in pigs with a diet-containing sodium n-butyrate. Acta Vet Hung.

[21] Fang CL, Sun H, Wu J, Niu HH, Feng J (2014). Effects of sodium butyrate on growth performance, haematological and immunological characteristics of weanling piglets. J Anim Physiol Anim Nutr (Berl).

[22] Canfora EE, Jocken JW, Blaak EE (2015). Short-chain fatty acids in control of body weight and insulin sensitivity. Nat Rev Endocrinol.

[23] Brumbaugh DE, Tearse P, Cree-Green M, Fenton LZ, Brown M, Scherzinger A, Reynolds R, Alston M, Hoffman C, Pan Z (2013). Intrahepatic fat is increased in the neonatal offspring of obese women with gestational diabetes. J Pediatr.

[24] Catalano PM (2010). The impact of gestational diabetes and maternal obesity on the mother and her offspring. J Dev Orig Health Dis.

[25] Berlanga A, Guiu-Jurado E, Porras JA, Auguet T (2014). Molecular pathways in non-alcoholic fatty liver disease. Clin Exp Gastroenterol.

[26] Hossain Z, MacKay D, Friel JK (2016). Fatty Acid Composition in Feeds and Plasma of Canadian Premature Infants. J Pediatr Gastroenterol Nutr.

[27] Bergman EN (1990). Energy contributions of volatile fatty acids from the gastrointestinal tract in various species. Physiol Rev.

[28] Lin Y, Fang ZF, Che LQ, Xu SY, Wu D, Wu CM, Wu XQ (2014). Use of sodium butyrate as an alternative to dietary fiber: effects on the embryonic development and anti-oxidative capacity of rats. PLoS ONE.

[29] Claus TH, Lowe DB, Liang Y, Salhanick AI, Lubeski CK, Yang L, Lemoine L, Zhu J, Clairmont KB (2005). Specific inhibition of hormone-sensitive lipase improves lipid profile while reducing plasma glucose. J Pharmacol Exp Ther.

[30] Robert Z, Strauss JG, Guenter H, Gabriele S, Ruth B-G, Monika R, Achim L, Georg N, Frank E, Albin H, Rudolf Z (2014). Fat Mobilization in Adipose Tissue Is Promoted by Adipose Triglyceride Lipase. Science.

[31] Chakrabarti P, Kim JY, Singh M, Shin YK, Kim J, Kumbrink J, Wu Y, Lee MJ, Kirsch KH, Fried SK, Kandror KV (2013). Insulin inhibits lipolysis in adipocytes via the evolutionarily conserved mTORC1-Egr1-ATGL-mediated pathway. Mol Cell Biol.

[32] Moon HS, Batirel S, Mantzoros CS (2014). Alpha linolenic acid and oleic acid additively down-regulate malignant potential and positively cross-regulate AMPK/S6 axis in OE19 and OE33 esophageal cancer cells. Metabolism.

[33] Wang A, Si H, Liu D, Jiang H (2012). Butyrate activates the cAMP-protein kinase A-cAMP response element-binding protein signaling pathway in Caco-2 cells. J Nutr.

[34] Su CL, Sztalryd C, Contreras JA, Holm C, Kimmel AR, Londos C (2003). Mutational analysis of the hormone-sensitive lipase translocation reaction in adipocytes. J Biol Chem.

[35] Fricke K, Heitland A, Maronde E (2004). Cooperative activation of lipolysis by protein kinase A and protein kinase C pathways in 3 T3-L1 adipocytes. Endocrinology.

[36] Diaz P, Harris J, Rosario FJ, Powell TL, Jansson T (2015). Increased placental fatty acid transporter 6 and binding protein 3 expression and fetal liver lipid accumulation in a mouse model of obesity in pregnancy. Am J Physiol Regul Integr Comp Physiol.

[37] Mennitti LV, Oliveira JL, Morais CA, Estadella D, Oyama LM, Nascimento CM O d, Pisani LP (2015). Type of fattyacids in maternal diets during pregnancy and/or lactation and metabolic consequences of theoffspring. J Nutr Biochem.

[38] McArthur MJ, Atshaves BP, Andrey F, Foxworth WD, Kier AB, Friedhelm S (1999). Cellular uptake and intracellular trafficking of long chain fatty acids. J Lipid Res.

[39] Petrescu AD, McIntosh AL, Storey SM, Huang H, Martin GG, Landrock D, Kier AB, Schroeder F (1831). High glucose potentiates L-FABP mediated fibrate induction of PPARalpha in mouse hepatocytes. Biochim Biophys Acta.

[40] Jan FC G, Van der Vusse GJ (1996). Cellular fatty acid-binding proteins:Their function and physiological significance. Prog Lipid Res.

[41] Goldstein JL, Brown MS (2009). The LDL receptor. Arterioscler Thromb Vasc Biol.

